# Layered Screening and Contact-Limiting Interventions Are Necessary to Reduce SARS-Cov-2 Outbreak Risks in Large Urban Jails

**DOI:** 10.4269/ajtmh.22-0716

**Published:** 2023-09-05

**Authors:** Krzysztof Sakrejda, Chad Zawitz, Robert A. Weinstein, William Trick, Joshua Rafinski, Kelly Broen, Hannah Steinberg, Kyle J. Popovich, Jon Zelner

**Affiliations:** ^1^ Department of Epidemiology, University of Michigan School of Public Health, Ann Arbor, Michigan;; ^2^ Center for Social Epidemiology and Population Health, University of Michigan School of Public Health, Ann Arbor, Michigan;; ^3^ Department of Medicine, Cook County Health, Chicago, Illinois;; ^4^ Division of Infectious Disease, Rush University Medical Center, Chicago, Illinois

## Abstract

Highly transmissible infections with short serial intervals, such as SARS-Cov-2 and influenza, can quickly overwhelm healthcare resources in institutional settings such as jails. We assessed the impact of intake screening measures on the risk of SARS-CoV-2 outbreaks in this setting. We identified which elements of the intake process created the largest reductions in caseload. We implemented an individual-based simulation representative of SARS-Cov-2 transmission in a large urban jail utilizing testing at entry, quarantine, and post-quarantine testing to protect its general population from mass infection. We tracked the caseload under each scenario and quantified the impact of screening steps by varying quarantine duration, removing testing, and using a range of test sensitivities. We repeated the simulations under a range of transmissibility and community prevalence levels to evaluate the sensitivity of our results. We found that brief quarantine of newly incarcerated individuals separate from the existing population of the jail to permit pre-quarantine and end-of-quarantine tests reduced SARS-CoV-2 caseload 30–70% depending on test sensitivity. These results were robust to variation in the transmissibility. Further quarantine (up to 14 days) on average created only a 5% further reduction in caseload. A multilayered intake process is necessary to limit the spread of highly transmissible pathogens with short serial intervals. The pre-symptomatic phase means that no single strategy can be effective. We also show that shorter durations of quarantine combined with testing can be nearly as effective at preventing spread as longer-duration quarantine up to 14 days.

## INTRODUCTION

As of July 2023, more than 640,000 incarcerated people had tested positive for SARS-CoV-2 in the United States, and more than 2,800 had died of COVID-19.^1^ In the earliest months of the COVID-19 pandemic, large outbreaks occurred in jails, prisons, and detention facilities. For example, in separate correctional facilities in Arkansas^2^ and Wisconsin,^3^ 80% of incarcerated individuals were infected with SARS-CoV-2 during just 2 months from March to April 2020. These high rates of transmission in detention facilities reflect the fact that incarcerated individuals often share close quarters,^4^ where rapid transmission combined with the short latent period of SARS-CoV-2 can result in difficult-to-control transmission, leading to large outbreaks. Other respiratory pathogens (e.g., influenza) that share the short latent period of SARS-CoV-2 and result in extremely heterogeneous outcomes have long caused significant epidemics^5^ in institutional settings, and the COVID-19 response provides an opportunity to evaluate broadly applicable control strategies.

The size of outbreaks and burden of severe disease and fatality in large urban jails have slowed since the earliest months of the pandemic. Nonetheless, these facilities remain vulnerable to changing epidemic conditions, such as the emergence of more-infectious variants^6^^,^^7^ that increase the rate of introduction and spread. Urban jails are porous institutions, characterized by relatively short duration of incarceration, contact with outside visitors, and daily movements of staff in and out of the facility. Because of the short average length of stay, jail-based transmission of SARS-CoV-2 may also pose risks to community contacts of incarcerated individuals, in an echo of earlier findings on the role of jails in community-based transmission of sexually transmitted infections,^8^^,^^9^ drug-resistant tuberculosis,^10^ and methicillin-resistant *Staphylococcus aureus*.^11^

During the initial wave of the COVID-19 pandemic in spring 2020, many facilities instituted intensive infection control measures that successfully reduced COVID-19 incidence. Guidance for correctional facilities from the CDC continues to focus on preventing the introduction of SARS-CoV-2 into facilities through a combination of intake testing and quarantining people after incarceration.^12^ These recommendations reflect the success of these measures for reducing the spread of SARS-CoV-2 in correctional facilities. For example, Zawitz et al.^13^ demonstrated that after implementation of intensive intake screening, quarantine, and isolation protocols, case rates in the Cook County Jail (CCJ) abruptly and dramatically fell, even as the rate of SARS-CoV-2 infection in the city of Chicago increased.

However, these measures are costly in terms of facility space and staff resources, may result in delays or gaps in routine medical care, and can negatively impact the social and emotional well-being of incarcerated people.^14^^,^^15^ As vaccination rates in the community and in-custody populations increased and case rates across the United States fell during summer 2021, many facilities began revisiting their strategies for preventing introduction and transmission of SARS-CoV-2. However, the emergence of the highly transmissible Omicron (B.1.1.529) variant and of its multiple more-infectious subvariants has highlighted the vulnerability of facilities employing less-intensive infection control procedures to sudden shifts in risk.

In this paper, we use a transmission model to examine the relative contributions of testing, quarantine, and isolation procedures on the burden of infection in a simulated urban jail facility. As changes in transmissibility, vaccination, and acceptance of infection controls have varied, the CCJ has continued to implement these procedures. Our goal is to determine which of these procedures is most critical for preventing the introduction and transmission of SARS-CoV-2 and which can safely be relaxed under different scenarios of community-level incidence (driving the rate of introduction) and transmissibility (driving the rate of transmission within the facility).

## MATERIALS AND METHODS

We developed a detailed stochastic simulation model of the intake screening, quarantine, and transmission process at the CCJ. We used this model to estimate the reduction associated with these layered interventions in the number of infections acquired by incarcerated individuals during their time in the facility. We counted all infections in the transmission chain. We then conducted a series of “knockout” simulation experiments in which specific mitigation measures were relaxed or eliminated. This allowed us to evaluate the relative importance of each measure to the reduction of risk among individuals in the CCJ. We also examined the robustness of these changes to variation in the intensity of infection in the community and the rate of transmission within the CCJ. In the following section, we outline the structure of our transmission model and data inputs to the model in detail.

### Transmission model.

**Figure 1.**
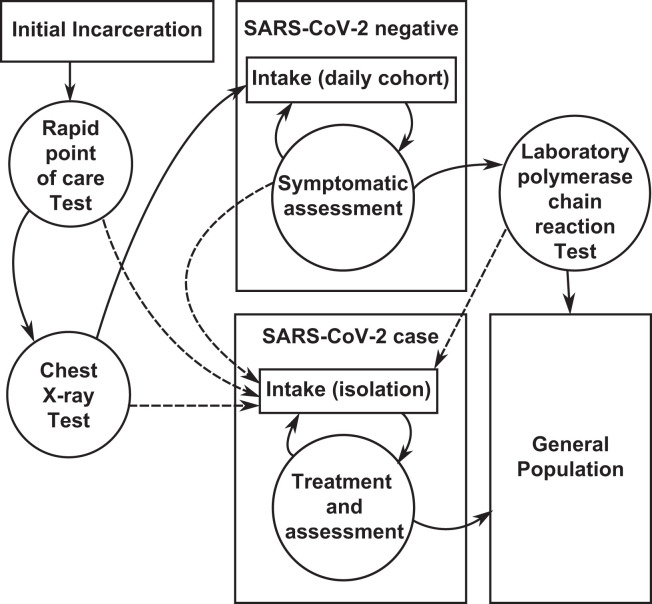
Flow diagram of the intake screening process for newly incarcerated people. This diagram represents the sequence of screening steps undertaken between an individual’s initial detention and his or her entry into the general population of the jail. Boxes in the figure represent stages of the intake screening process, conditional on an individual’s infection state as measured by viral testing and symptomatic screening. Circles represent specific screening steps used. Solid lines indicate the most common transitions in the model, whereas dashed lines indicate a subset of transitions between states experienced only by individuals who tested positive for SARS-CoV-2.

Transmission of SARS-CoV-2 was modeled using a modified discrete-time stochastic Susceptible-Exposed-Infectious-Recovered (SEIR) model.^16^ Individuals in the susceptible (S) state are assumed to have no natural or vaccine-derived immunity to infection. The exposed (E) group includes individuals in the 2- to 11-day latent period^17^ during which they cannot transmit and their infection cannot be detected by polymerase chain reaction (PCR) testing or symptomatic surveillance. The infected (I) group includes symptomatic and asymptomatically infectious individuals who can transmit disease and are likely to return a positive PCR test result. Those who are detected are assumed to be isolated (Figure 1). Finally, the recovered (R) group includes individuals who have recovered from the infection and are assumed to be immune. All transition times are exponentially distributed to coarsely approximate the course of infection. We did not consider the possibility of reinfection, as our focus is on the intake cohorts, which are typically cleared within 14 days. Administrative processes were simulated separately from infection so that an individual in any given compartment could pass their simulated “duration of detention” and move to a separate absorbing state (where they keep their final status but can no longer transmit infection).

### Intake, screening, quarantine, and release.

At the beginning of each simulated day, individuals are incarcerated at the facility and subject to a series of infection control procedures. After initial rapid PCR testing and isolation of cases identified by testing or symptomatic screening, all individuals admitted on a given day who were not flagged for isolation are placed in a quarantine cohort. We assumed that this cohort is well mixed (i.e., all individuals in the cohort have equal contact with each other) but that these individuals do not have contact with anyone else in the facility. After quarantine—which is varied from 2 to 14 days across scenarios—individuals who obtain a negative PCR result are sent to the general population of the facility, which is assumed to be well mixed. At any point, individuals can leave the quarantine or general jail population if they are transferred to prison or released to the community. We simulated the duration of incarceration for each individual from an exponential distribution. with a rate matching the observed duration of detention in CCJ data (Table 1) and capped at 30 days maximum.

**Table 1:** Key model parameter definitions, values, and sources

Parameter	Description	Value	Source
Latent period (1/σ)	Average time interval between infection and first possible transmission; during this period, the individual is not capable of infecting others	5 Days	Kissler et al.,^18^ Lauer et al.,^17^ Singanayagam et al.,^19^ Nishiura et al.^20^
Infectious period (1/γ)	Average time interval during which the individual is capable of infecting others	14 Days	Kissler et al.,^18^ Nishiura et al.,^20^ Singanayagam et al.^19^
Fully asymptomatic	Proportion of infections that never develop symptoms	20%	Kissler et al.,^18^ Mizumoto et al.^21^
Reproduction number (*R*_0_)	Expected number of secondary infections per incident case in an otherwise fully susceptible population	1.1, 3, 5	Vissat et al.^22^
Intake community prevalence	SARS-CoV-2 prevalence (pre-infectious and infectious) in the intake population	1%, 10%	CCJ data on COVID-19 testing at intake
Intake rate	Number of people incarcerated each day	100	CCJ data on daily intake
Duration of detention (1/κ)	Scale for the exponential distribution describing how long individuals are incarcerated	30	CCJ data on individual duration of incarceration

CCJ = Cook County Jail.

### Latent period.

We chose input parameter ranges that are consistent with estimates from the clinical literature on SARS-CoV-2 infection and testing. Studies of SARS-CoV-2 transmission show that the latent period and the incubation period are approximately the same duration,^17^^–^^19^ starting at 2 days, with most infections that will ultimately become symptomatic developing symptoms within 10 days. The serial interval between symptomatic infections^20^ closely tracks the expected serial interval based on quantitative PCR and viral culture data.^18^^,^^19^ Both show that highest infectivity occurs in the first week after symptoms develop and decays over up to 3 weeks.^18^

### Rate of SARS-CoV-2 introduction.

A critical driver of infection within the CCJ is the rate of introduction of infected individuals from the community into the facility. To characterize this, we used administrative data to calculate an intake rate of approximately 100 individuals per day and assumed individuals entered the facility at a uniformly distributed time point within their infectious window. We varied the rate of infection among newly incarcerated people from a low value (1%) to a high value (10%). These two scenarios matched the extremes of community prevalence seen in data from CCJ intake testing.

### Simulation strategy.

Each simulation was run for 30 days, with the outcome measure of interest being the total number of individuals who were admitted to the facility during this period who were infected with SARS-CoV-2 during the 30-day period. This facilitated straightforward comparison of the relative impact of different screening and isolation measures on risks between scenarios.

For each set of parameters, we generated 100 simulation replicates and recorded the number of new infections generated within the jail population over the 30-day simulation period. We included secondary infections and longer transmission chains within the facility but excluded individuals who were infectious at intake. Depending on the type of test(s) used or the combination of tests and screening procedures, the sensitivity of the screening process could vary widely as a function of the test kit used and the currently circulating variant. As a result, rather than assigning sensitivities to tests, we evaluated the effect of intake and end-of-quarantine (EOQ) tests with sensitivities of 0.4, 0.6, 0.85, and 1. We simulated all combinations of these conditions for low (1%) and high (10%) community prevalence under low (*R*_0_ = 1.1), medium (*R*_0_ = 3), and high (*R*_0_ = 5) rates of within-facility transmission with quarantine durations of 2, 4, 7, and 14 days as well as an indefinite intake cohort quarantine. See Supplemental Appendix A for a discussion of parameter selection and Table 1 for the specific ranges of transmission parameter values explored in simulations as well as the range of sensitivities for each screening method. For more details on how the range of sensitivity values for each screening step was obtained, see Supplemental Appendix A.

## RESULTS

### No-intervention baseline.

Over 30 simulation days, approximately 3,000 individual incarceration histories were simulated, with the simulated standing population starting at zero and rising to ∼2,000 over the first 30 days and then remaining stable. In the absence of screening or intake quarantine, all results were as expected, with higher prevalence at intake (1% versus 10%) and increasing values of *R*_0_ associated with increased incidence. Baseline results are presented as observed counts of infections and percentage of the 30-day incarcerated population infected. For a very low infectivity scenario (*R*_0_ = 1.1), the total expected number of jail-acquired infections approximately matched the number of imported community infections regardless of quarantine duration. At a higher infectivity (*R*_0_ = 3), new infections increased to 289 (10%) new infections at 1% community prevalence and 1,127 (38%) new infections at 10% community prevalence. Further increases in infectivity (*R*_0_ = 5) began to deplete the available susceptible population, resulting in 1,148 (38%) new infections based on 1% community prevalence and 1,867 (62%) new infections at 10% community prevalence.

### Impact of intake quarantine duration.

**Figure 2.**
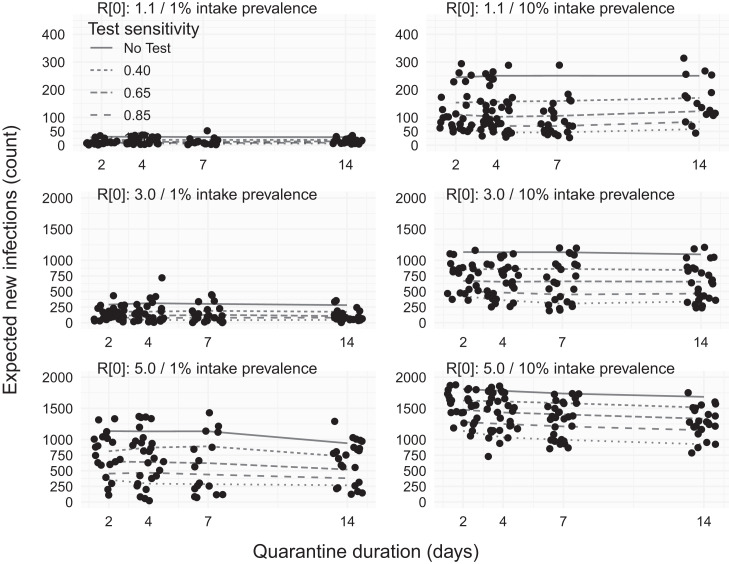
Expected number of in-jail infections for varying sensitivities of intake and end-of-quarantine testing. Rows indicate different rates of within-facility transmissibility, increasing from low-transmissibility (*R*_0_ = 1.1) to high transmission (*R*_0_ = 5) from top to bottom. Columns indicate different levels of prevalence among individuals admitted to the facility, showing both the low-prevalence (1%, left) and high-prevalence (10%, right) scenarios. Points are a subsample of incidence from individual simulation replicates, and lines in each panel show the association between quarantine duration (2, 4, 7, or 14 days) and the expected number of within-facility infections (*y* axis). Solid lines show the relationship between quarantine duration and incidence under a control scenario in which there is no intake or end-of-quarantine testing. Dashed and dotted lines indicate different sensitivities of both the intake and end-of-quarantine test.

At low infectivity (*R*_0_ = 1.1), intake cohort quarantining did not have a meaningful effect on the number of new infections compared with scenarios in which no cohorts were used. At moderate infectivity (*R*_0_ = 3), intake cohort quarantine up to 14 days also had little effect. At high infectivity (*R*_0_ = 5), the effect of quarantine duration on within-facility risk was more pronounced. There was not a meaningful difference associated with quarantine durations less than 7 days compared with none. But a reduction from 1,148 (38%) to 941 (31%) infections under a 14-day quarantine was observed (Figure 2, bottom left) in the low community-prevalence scenario (1%), and a reduction from 1,867 to 1,592 expected infections was observed (Figure 2, bottom right) in the high community-prevalence scenario (10%). At 1% intake prevalence, extending quarantine until release (not shown) resulted in a more dramatic reduction from 1,148 (38%) to 490 (16%) infections. At high levels of community prevalence, even this indefinite quarantine resulted in only a moderate improvement, reducing 1,867 (62%) infections to 1,592 (53%) infections, reflecting frequent introduction of incubating disease under this scenario. For all settings and quarantine durations, the SD of the expected case count was not affected by quarantine.

### Testing impact.

The results for impacts of testing presented below are always defined with reference to a comparison that includes the same duration of quarantine so that they represent the value added by the test in the given scenario. In more challenging scenarios with higher *R*_0_ values and higher intake prevalence, the tests always capture a higher number of infections proportional with test sensitivity, and we present primarily the percent reduction relative to the expected average number of infections.

### Efficacy of combined intake and EOQ testing.

At low transmission (*R*_0_ = 1.1) and low intake prevalence (1%), combining intake and EOQ testing could reduce jail-acquired infections 33–39% with a worst-case, low-sensitivity (0.4) test and 76–84% for a perfect test, with quarantine duration having little effect (Figure 2, top four panels). At high intake prevalence, quarantine duration still had little effect, and relative reductions in new infections were similar as a function of test sensitivity. The results were similar at moderate transmission (*R*_0_ = 3) and low intake prevalence; however, at high intake prevalence, a low-sensitivity test only reduced infections 23–25% and a perfect test reduced new infections 62–73% (Figure 2, center row, right panel). High transmission (*R*_0_ = 5.0) combined with low prevalence produced similar results, whereas high transmission and high prevalence led to reduced effectiveness, with only a 9–10% reduction for low-sensitivity testing and a 37–45% reduction with a perfect test (Figure 2, bottom right panel).

### Impact of removing EOQ testing.

**Figure 3.**
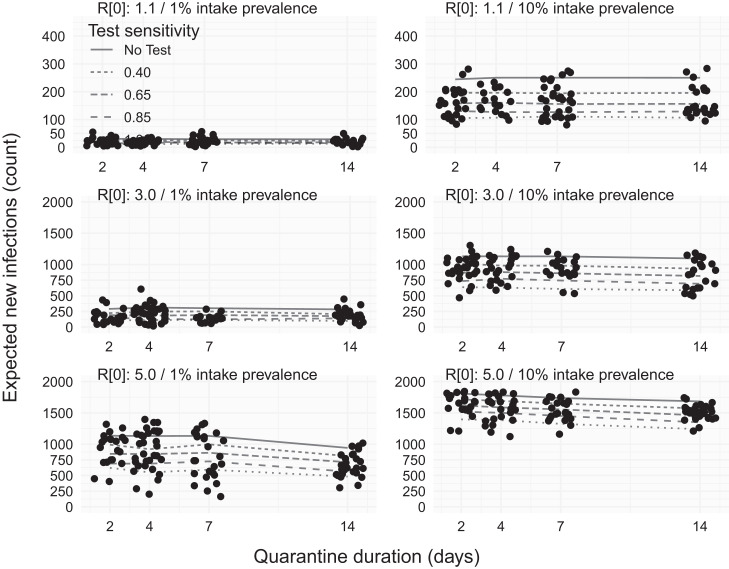
Expected number of in-jail infections with only intake testing and no end-of-quarantine testing. Rows indicate different rates of within-facility transmissibility, increasing from low transmissibility (*R*_0_ = 1.1) to high transmission (*R*_0_ = 5) from top to bottom. Columns indicate different levels of prevalence among individuals admitted to the facility, with low-prevalence (1%, left) and high-prevalence (10%, right) scenarios. Points indicate (jittered) incidence from a subsample of individual simulation replicates, and lines in each panel show the association between quarantine duration (2, 4, 7, or 14 days) and the expected number of within-facility infections (*y* axis). Solid lines show the relationship between quarantine duration and incidence under a control scenario in which there is no intake or end-of-quarantine testing. Dashed and dotted lines indicate different sensitivities of the intake test.

At low infectivity, screening using only an intake test with 40% sensitivity resulted in a 15–20% reduction in infections in the low community-prevalence scenario and a 20–22% reduction in within-facility infections in the high community-prevalence scenario. Quarantine duration had little effect on overall case rates when intake testing was used, with reductions driven primarily by test sensitivity. For example, an ideal 100% sensitive test was associated with a reduction of 54–62% in infections regardless of intake prevalence (Figure 3).

At moderate (*R*_0_ = 3) infectivity, a low (0.45) sensitivity test led to a 17–27% reduction in new infections under low prevalence and 11–15% under high prevalence (Figure 3, top row). The effect of quarantine duration was still negligible, whereas higher test sensitivity was more important, with 62–65% reductions from a perfect test under low intake prevalence (Figure 3, center row, left). At high intake prevalence, even a perfect test only reduced new case incidence by 44–46% (Figure 3, center row, right). At high (*R*_0_ = 5.0) infectivity, the value of low-sensitivity (0.45) intake testing was limited, with only a 13–19% reduction under low prevalence and a 5–7% reduction under high prevalence. A perfect test reduced new case counts by 45–51% at low prevalence but only by 22–26% at high prevalence (Figure 3, bottom row).

### Impact of removing intake testing.

**Figure 4.**
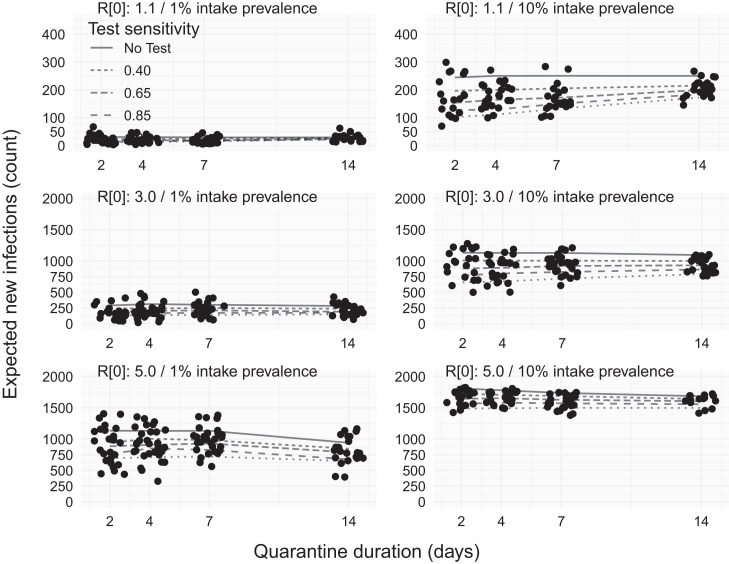
Expected number of in-jail infections with only end-of-quarantine testing and no intake testing. Rows indicate different rates of within-facility transmissibility, increasing from low transmissibility (*R*_0_ = 1.1) to high transmission (*R*_0_ = 5) from top to bottom. Columns indicate different levels of prevalence among individuals admitted to the facility, with low-prevalence (1%, left) and high-prevalence (10%, right) scenarios. Points indicate (jittered) outcomes from subsamples of individual simulation replicates, and lines in each panel show the association between quarantine duration (2, 4, 7, or 14 days) and the expected number of within-facility infections (*y* axis). Solid lines show the relationship between quarantine duration and incidence under a control scenario in which there is no intake or end-of-quarantine testing. Dashed and dotted lines indicate different sensitivities of the end-of-quarantine test.

The reduction in caseload associated with only using EOQ testing was strongly related to the duration of quarantine, with the effect of EOQ testing most pronounced for high-sensitivity tests and increased quarantine duration counterintuitively associated with increased caseload for low-sensitivity tests (Figure 4). This contrasts with intake testing, where the impact was not dependent on the quarantine duration. At low transmission and community prevalence, a low-sensitivity (0.40) EOQ test could reduce transmission 26% below what was achievable with a 2-day quarantine period alone but only 6% below the expected case counts for a 14-day quarantine period. When intake prevalence was high, the reduction in transmission ranged from 20% (2-day quarantine) to 14% (14-day quarantine) for low-sensitivity testing. These effects were more pronounced as test sensitivity increased. Under low intake prevalence, a perfect test reduced new infections by 63% relative to a 2-day quarantine, but only 24% relative to a 14-day quarantine. Under high prevalence, the impact of a perfect test was similar, ranging from a 58% reduction in new infections to a 31% reduction depending on the duration of quarantine.

## DISCUSSION

Our results highlight why employing multiple screening and infection control modalities is essential to slowing the introduction and spread of infections such as SARS-CoV-2 in large urban jails like the CCJ. Even in simulation scenarios where the sensitivity of intake diagnostic tests was assumed to be perfect, regular within-jail transmission was evident in our simulations, particularly when the within-jail transmission parameter *R*_0_ was high and SARS-CoV-2 infection was highly prevalent in the community. Our results clearly indicate the importance of maintaining a combination of non-pharmaceutical interventions (NPIs), for example, intake quarantine cohorts, in addition to a robust intake testing regimen for limiting transmission in these scenarios.

An important implication of our findings relates to the lower-than-expected impact of the elimination of very high sensitivity tests on the burden of infection in the facility. Varying the sensitivity of these tests between optimistic (i.e., 100%) and more-pessimistic estimates of their sensitivity ultimately had limited impact on incidence, with a perfect test reducing infections 68–79% under most combinations of prevalence and transmission with reductions in the worst scenario (*R*_0_ = 5, intake prevalence = 10%) limited to 42%. These findings could be explained by infected individuals who were not yet infectious, that is, not actively shedding virus. They will not be detected by any available screening and will not usually be detected in a timely fashion in the absence of regular testing with at least moderate sensitivity. In this case, testing must be combined with non-pharmaceutical, contact-limiting interventions such as quarantine, masking, and air quality improvements, which will reduce transmission throughout the facility. In fact, our results show that using a brief quarantine sandwiched between two tests reduced caseloads 30–70% (depending on test sensitivity). Extending quarantine up to 14 days created only ∼5% additional reductions in caseloads when averaged across scenarios. Although our simulations do not suggest that NPIs can eliminate the risk of introduction into the general population and do not directly address the impact of vaccination on this risk, their role in reducing the number of susceptible individuals reachable by infections not detected during the intake screening and testing steps is clear.

It would be a mistake, however, to interpret these results as pessimistic about the role of regular testing as a means of preventing the introduction and spread of SARS-CoV-2 in settings like the CCJ. Instead, a glass half-full view of these results suggests that it may be worthwhile to focus on the deployment of more frequent testing of individuals, both during a quarantine period and after release into the general population, as an additional line of prevention. Future studies should examine whether the frequent use of less-sensitive but more economical lateral-flow screening tests^23^ or pooled approaches, such as wastewater surveillance, may be a more effective use of testing resources than current practices.

Although our simulations faithfully represent screening and quarantine processes at the CCJ, they include important simplifying assumptions. Most notably, we assumed a fully susceptible population at the beginning of each simulation without infection- or vaccine-derived immunity. In addition, we did not account for heterogeneity in the infectiousness or susceptibility due to health behaviors, such as variable mask usage or variation in the intensity of viral shedding. We also did not examine the role of introductions of infection from the community into quarantine cohorts or the general population by staff members. These were intentional choices made to focus attention on the relative importance of different steps of the intake process in preventing the introduction and spread of a highly infectious respiratory pathogen such as SARS-CoV-2 in a large, high-turnover, urban jail like the CCJ. Lacking data on true levels of infection, we did not attempt to calibrate our simulation to observed data, and we summarized many details into the *R*_0_ values. Qualitatively, larger *R*_0_ values produce a process that results in very high level (> 50%) of infection in all cohorts, which matches experience on the fast spread of respiratory pathogens in jail settings.

Novel infections are a long-term challenge for congregate facilities such as the CCJ: SARS-CoV-2 variants continue to emerge, demonstrating increasing levels of immune escape, and monkeypox was recently identified within the CCJ.^24^ These continuing risks underscore the importance of an aggressive, multilayered approach for preventing the introduction and spread of novel SARS-CoV-2 variants and other emerging pathogens, such as monkeypox, in transmission-rich, high-turnover settings such as jails. Our results suggest that contact-limiting NPIs are a crucial adjunct to testing in such venues, particularly at times when community prevalence and risk of variant introduction is high. If infection rates are not controlled, high turnover will ensure that new infections seed cases in the broader community, making the implementation of better control strategies key to overall infection control. Future studies should examine whether an adaptive approach in which quarantine and testing regimens are modified based on measures of community- and facility-level prevalence can provide necessary prevention without depleting medical resources that could be devoted to other aspects of healthcare during incarceration.

## Supplemental Materials


Supplemental materials
