# Supplementary material for: Layered Screening and Contact-Limiting Interventions Are Necessary to Reduce SARS-Cov-2 Outbreak Risks in Large Urban Jails
Source: Am J Trop Med Hyg. 2023 Sep 5;109(4):874–80. doi: 10.4269/ajtmh.22-0716 (PMC10551074; doi:10.4269/ajtmh.22-0716)
Supplement: Supplementary file 1 [file tpmd220716.SD1.pdf]

Appendix A

Test Sensitivity Supplement

Test sensitivity is generally described as the proportion of cases detected out of some known number of cases. The lab sensitivity and specificity of Nucleic Acid Amplification Tests (NAATs) with repeat temperature-controlled amplification cycles is very nearly 100% for a known and sufficient amount of RNA in the test sample. This has resulted in its use as a “gold standard” for benchmarking other tests. In real world applications the true sensitivity of NAATs can vary due to sampling technique and infection dynamics which result in a window of high sensitivity for the commonly used on a naso-pharygeal sample. When used in contact tracing and testing individuals following known exposure, the test can be timed to coincide with this window (3-5 days following infection) resulting in real-world performance close to 100%. In other contexts this strategy is not always available and the real-world sensitivity of NAATs can be very variable. As a result, much of the literature on sensitivity for *all* COVID-19 laboratory tests is difficult to interpret.

To resolve this confusion we define the sensitivity precisely for our simulation as **the proportion of infectious individuals detected out of the total number of infectious individuals.** This results in a sensitivity of zero for all cases in the pre-infectious “exposed” category but it is consistent with the real world mechanistic evidence for PCR sensitivity as a function of RNA availability in real world infections that were tracked by exhaustive sampling of a finite population[1].

The real-world setting closest to our definition is when PCR sensitivity is defined as **the proportion of cases detected by NAAT at a fixed timepoint out of the total number of retrospectively identified cases**. A few studies have used this definition to show that the common naso-pharyngeal swab specimens show a sensitivity approximately 60%[2,3]. Further studies listed in a supplement to the Infectious Disease Society of America diagnostic guidelines (IDSA) on upper respiratory tract samples (supplemental figures, section E, figure s4) suggest a sensitivity of 40-60% while two studies which **did not** use the retrospective case discovery suggest higher sensitivity[4].

Many studies track only relative sensitivity as the proportion of individuals detected by a standard NAAT out of the total number of individuals detected by **any method**. These studies show very high sensitivity for NAAT (>90%), as NAAT is the most sensitive method and **if** a case is captured by **any** method, it is likely to be captured by NAAT. These studies are likely only useful for us in calculating the relative sensitivity of NAAT methods that use fewer amplification cycles, a single amplification cycle, or simply an alternative antigen-based method. According to the IDSA diagnostic guidelines supplement, single-cycle isothermal NAAT tests show (supplemental figures, section G, figure s8a) ~75% sensitivity relative to standard NAAT[4]. The final sensitivity of single-cycle isothermal NAAT tests is therefore roughly 0.6 (standard NAAT sensitivity) x 0.75 (relative single-cycle NAAT sensitivity) = 0.45 or 45% sensitivity.

References

1. Kissler, S. M. *et al.* Viral dynamics of acute SARS-CoV-2 infection. 2020.10.21.20217042 Preprint at https://doi.org/10.1101/2020.10.21.20217042 (2021).
2. Wang, W. *et al.* Detection of SARS-CoV-2 in Different Types of Clinical Specimens. *JAMA* **323**, 1843–1844 (2020).
3. Vandenberg, O., Martiny, D., Rochas, O., van Belkum, A. & Kozlakidis, Z. Considerations for diagnostic COVID-19 tests. *Nat. Rev. Microbiol.* **19**, 171–183 (2021).

4. Hanson, K. E. *et al.* Infectious Diseases Society of America Guidelines on the Diagnosis of Coronavirus Disease 2019. *Clin. Infect. Dis.* ciaa760 (2020) doi:10.1093/cid/ciaa760.

Parameter Selection

Test Sensitivity Parameter Selection: Real-world sensitivity of antibody tests, temperature-cycled PCR, and isothermal PCR tests all vary widely primarily due to the timing of the test with respect to the timing of the viral load in individuals and the sampling site. In a surveillance setting the quality control for sample collection can also be critical and the timing of testing with respect to time since exposure is typically unknown. We cover the entire range of sensitivities we have seen reported in different scenarios and extend the range to 100% in order to evaluate the importance of high-sensitivity tests relative to the overall screening strategy.

Community Prevalence: administrative data from the Cook County Jail shows that between mid-June 2020 and mid-February 2021 the positivity rate of tests administered on intake ranged between 1% and 9%. We used 1% and 10% community prevalence to span this range and describe the intensity of infection seeding into the facility. The primary qualitative difference is that at 1% it is common for daily intake groups to have zero or one infections so that high-quality screening can effectively delay seeding of infections into the facility. Near 10% days with 0-1 cases are rare screening primarily functions to reduce the **number** of infections entering the facility rather than modifying the **timing** of entry.

Transmission: in a crowded jail setting SARS-CoV-2 can have a very high R0 value, and with combinations of movement restrictions, mask wearing, population reductions combined with single-celling, testing, quarantine, and isolation, and other standard procedures the Cook County Jail demonstrated the ability to end epidemics even when community levels remained high. To bracket these possibilities we used R0 values of 1.1, 3, and 5. While values higher than 5 are plausible, such high transmission scenarios in our simulation result in infections being limited by exhausting the pool of susceptible individuals and are not consistent with infection control under any intake testing scenario.

References

1. Zawitz, Chad, Sharon Welbel, Isaac Ghinai, Connie Mennella, Rebecca Levin, Usha Samala, Michelle Bryant Smith et al. "Outbreak of COVID-19 and interventions in a large jail—Cook County, IL, United States, 2020." *American Journal of Infection Control* 49, no. 9 (2021): 1129-1135.
